# Surgical management and results of glenohumeral combination fractures of the anterior glenoid rim and the proximal humerus

**DOI:** 10.1007/s00402-024-05577-y

**Published:** 2024-10-02

**Authors:** Eileen Kerkhoff, Christopher Ull, Valentin Rausch, Maria Alexandra Bernstorff, Dominik Seybold, Thomas Armin Schildhauer, Matthias Königshausen

**Affiliations:** Berufsgenossenschaftliches Universitätsklinikum Bergmannsheil, Bürkle de La Camp-Platz 1, 44789 Bochum, Germany

**Keywords:** Glenoid fracture, Bony bankart lesion, Glenoid rim fracture, Proximal humerus fracture, Glenohumeral combination fracture

## Abstract

**Introduction:**

The combination of anterior large glenoid rim fractures (GRF) and proximal humerus fractures (PHF) is rare, with limited data available on specific treatments for these glenohumeral combination fractures (GCF). This study aimed to evaluate the treatment approaches for GCF, analyze patient outcomes, and outline surgical management strategies for different fracture types.

**Materials and methods:**

This retrospective study included patients with GCF, excluding those with fossa glenoidalis fractures, isolated greater tuberosity fractures, or small glenoid rim fractures (< 5 mm). Preoperative radiographs, CT scans, and follow-up radiographs were reviewed. Clinical outcomes were assessed using the Constant-Murley Score (CMS), Western Ontario Shoulder Instability Index (WOSI), Rowe Score (RS), and Oxford Shoulder Score (OSS).

**Results:**

Sixteen patients with 17 GCFs (mean age 62 years) were followed for an average of 39 months. PHFs were categorized into three-part (76%), four-part (12%), and two-part fractures (12%). The average medial displacement of GRF was 5 mm, with an average dehiscence of 4 mm in the sagittal plane. Fourteen patients (88%) underwent surgical treatment; 35% had only the PHF surgically addressed, while 53% had both lesions surgically treated. Two patients (12%) received non-operative treatment. Complications were observed in 29% of cases, primarily involving the humeral side. The average CMS was 68 points, WOSI was 75%, RS was 77 points, and OSS was 41 points.

**Conclusion:**

Treating GCF is complex and routinely necessitates surgical intervention, with or without GRF refixation. CT imaging is crucial for precise assessment of fracture morphology. The involvement of the minor tuberosity is critical in selecting the optimal surgical approach and managing the subscapularis muscle.

## Introduction

**Fig. 1:**
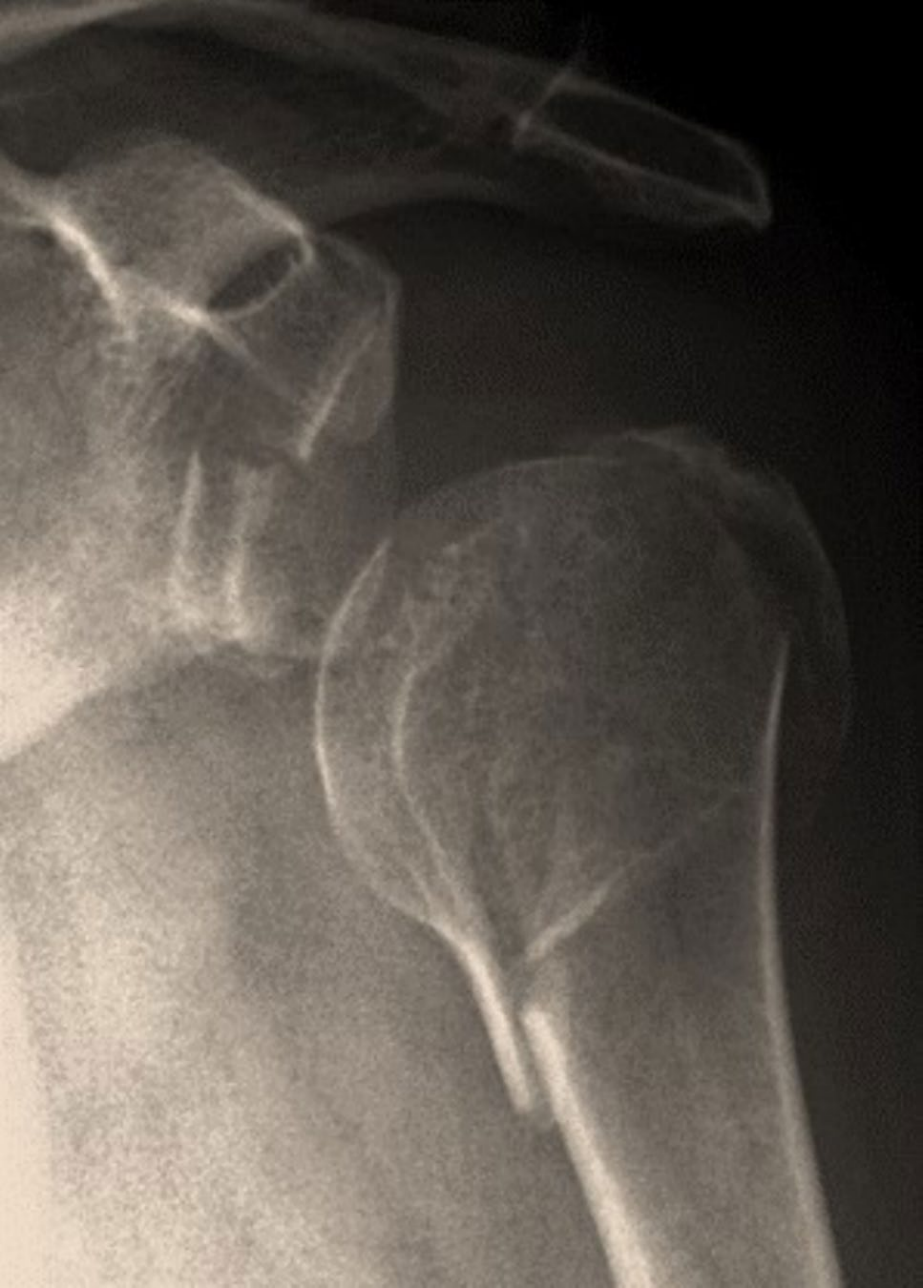
Glenohumeral combination fracture (proximal humerus and anterior glenoid rim fracture, a.p.-radiograph)

The combination of proximal humerus fractures (PHF) and large anterior glenoid rim fractures (GRF) represents a rare injury pattern referred to as glenohumeral combination fractures (GCF) (Fig. 1). These severe shoulder injuries are first described within a generalized classification of scapula fractures as Euler and Rüedi type E fractures [1]. A more detailed classification exclusively for glenohumeral combination fractures was introduced in 2019 by Königshausen et al., categorizing these injuries based on their severity. In this classification, the combined fracture of the glenoid rim and the proximal humerus is identified as type 2, as outlined in Table 1 [2].

**Table 1:** Classification of glenohumeral combination fractures [2]

Type	Classification of glenohumeral combination fractures
Type 1a	Large glenoid rim fracture (GRF) AND greater tuberosity fracture (GTF)
Type 1b	Type 1a with additional coracoid process fracture (CPF)
Type 2	Large glenoid rim fracture (GRF) AND proximal humerus fracture (PHF)
Type 3	Glenoid fossa fracture (GFF) AND greater tuberosity fracture (GTF)
Type 4	Glenoid fossa fracture (GFF) AND proximal humerus fracture (PHF)

GRFs should be distinguished from avulsion injuries of the anterior glenoid rim, also known as “chip fractures” (Ideberg 1A). Fractures of the anterior glenoid measuring ≥ 5 mm are classified as Ideberg 1B lesions and correspond to GRF or large “bony Bankart lesions” [3]. GRF are more frequent in a middle-aged population [3–5]. For isolated GRFs, two trauma mechanisms are typically considered: (1) the more common shoulder dislocation with an associated Hill-Sachs lesion (HSL) and (2) a presumed direct impact on the shoulder resulting in subluxation and subsequent force transmission to the anterior glenoid rim [5]. Numerous studies have been published on the reconstruction of isolated proximal humerus fractures or isolated fractures of the anterior glenoid rim [3, 4, 6–29]. However, to our knowledge, specific therapy recommendations for the rare combined injuries of the proximal humerus and the anterior glenoid rim are limited to a few case reports. These reports predominantly describe the combination of anterior glenoid rim fractures and isolated greater tuberosity fractures, with a primary focus on the outcome of the glenoid fracture [11, 13, 29–32]. As far as we know, no published studies exist on outcomes of reconstructed (ORIF) type 2 glenohumeral combination fractures within the literature. Therefore, this study aimed to evaluate the clinical outcomes of rare GCF (PHF and GRF, type 2) and discuss therapeutic strategies.

## Patients and methods

Patients with a GCF were identified retrospectively using the clinic’s internal database, including patients aged 18 years or older. Ethical approval was obtained from the local ethical board. After a postal invitation, informed consent was obtained.

Inclusion criteria were combination fractures of the humeral head and GRF (type 2; Table 1 [2]), treated either with surgical reconstruction via ORIF or non-operatively. We excluded GCF-cases treated with reverse shoulder arthroplasty or hemiarthroplasty, as well as GRF combined with isolated greater tuberosity fractures (type 1).

Preoperative imaging, including anteroposterior [a.p.] and axial view (or Y view) radiographs and CT scans, were evaluated. The fragment dislocation of the GRF was measured in the coronal and sagittal planes. PHF were classified according to the AO and Resch et al. classifications [33]. Two experienced surgeons and one radiologist analyzed further control radiographs, specifically assessing for avascular necrosis, osteoarthritis (classified according to Samilson and Prieto [34]), secondary displacement, or non-union.

Before the indication of the surgery, a precise preoperative CT evaluation of the fracture morphology was performed, particularly to identify fracture types involving the minor tuberosity, which significantly influenced our treatment decisions. Indications for the surgical treatment of the PHF were displacement of the tuberosities of more than 1 cm, cranial displacement of the major tuberosity > 5 mm as well as a tilt of the humeral head > 20° (Neer criteria) [35]. Indications for GRF treatment remain unclear in the literature and were individually assessed by the treating surgeon. Generally, indications include GRFs including more than 20% anterior glenoid involvement and a significant step-off of at least 3–4 mm. Centricity of the humeral head, typically used for GRF indications [4, 5, 36], was not considered a criterion due to the secondary importance of the fractured and displaced humeral head.

For the evaluation of the clinical outcome, we used one objective (Constant-Murley Score (CMS), with external rotation in degrees)), one mixed (Rowe Score (RS)) and two self-assessment scores (Western Ontario Shoulder Instability Index (WOSI), Oxford Shoulder Score (OSS)). Shoulder stability was clinically assessed using the apprehension test at 90° of abduction.

## Surgical procedure

General anesthesia and antibiotic prophylaxis were used in every patient. In the beach chair position, a deltopectoral approach was chosen in all surgical cases. Two main strategies were employed when surgery was indicated for both lesions (PHF and LGRF):

For PHF cases involving the minor tuberosity, the GRF was addressed first by opening the rotator interval and retracting the minor tuberosity. The GRF was then refixed using anchors or cannulated screws. Following this, the PHF, including the minor tuberosity, was reduced and stabilized with locking plate osteosynthesis.

**Fig. 2:**
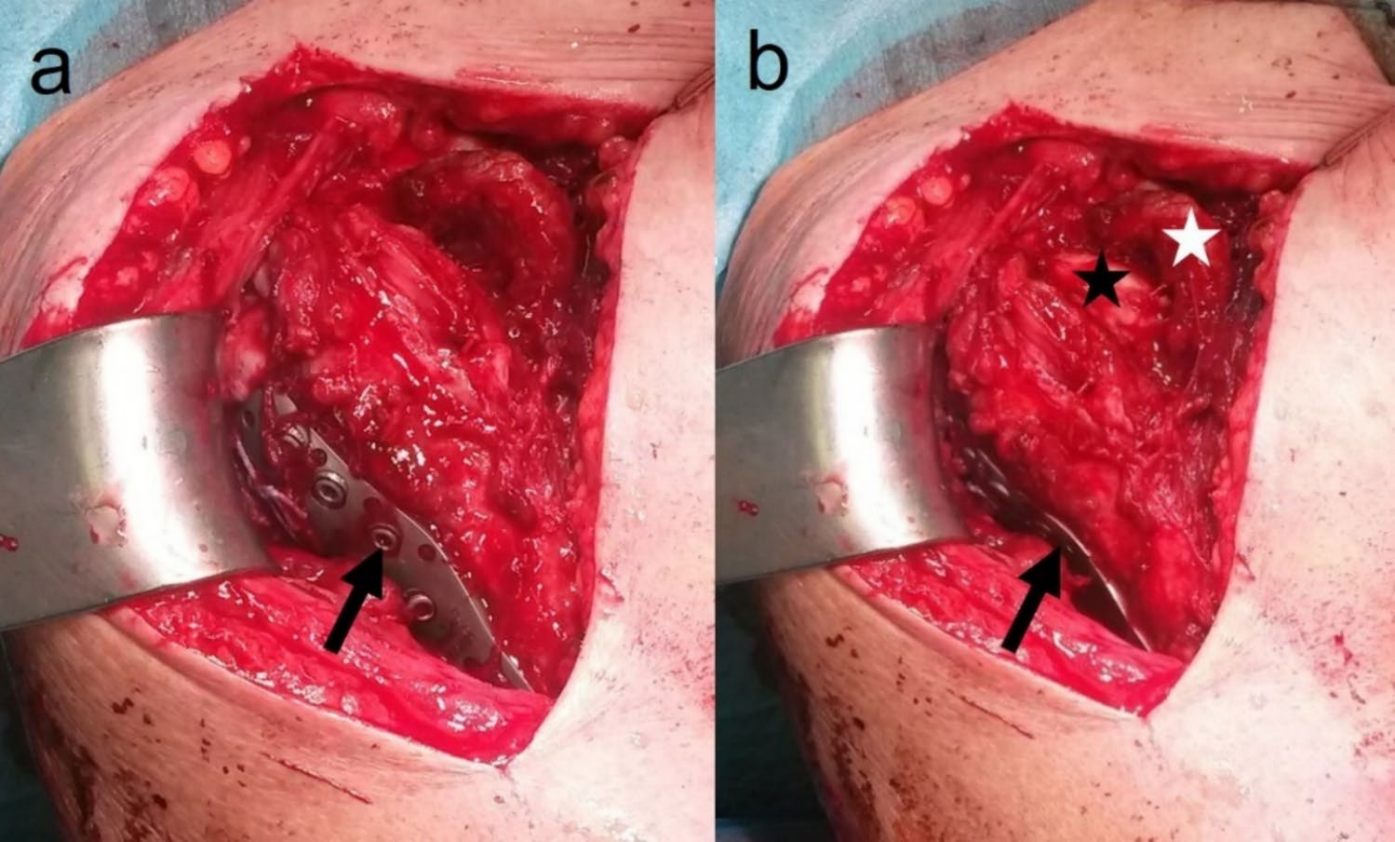
demonstrates the operative strategy for glenohumeral combination fractures (proximal humerus and anterior glenoid rim fracture) if there is an indication for surgery for the glenoid rim in addition to the proximal humerus. In case of an intact minor tuberosity (2- or 3-part fracture): First, the proximal humerus is fixed using locking plate osteosynthesis (arrow: plate). Second, the subscapularis muscle tendon is tenotomized (alternatively splitted) (b, the arm in more external rotation, arrow: plate, white asterisk: tendon; black asterisk: cartilage of humeral head) for fixation of the glenoid rim. In case of a 4-part fracture involving the minor tuberosity, the glenoid rim should be fixed primarily via taking down the minor tuberosity medially to remain the subscapularis tendon intact. Afterwards, the proximal humerus (including the minor tuberosity) should be fixed

In cases without minor tuberosity involvement, the PHF was addressed first with locking plate osteosynthesis. Subsequently, a subscapularis muscle (SSC) tenotomy, or alternatively an SSC-split approach, was performed to access the anterior glenoid rim, where the GRF was then fixed with anchors or cannulated screws (Fig. 2). For optimal shoulder function, the subscapularis tendon was then securely fixated.

In all surgeries involving the glenoid, a long head of the biceps (LHB) tenotomy was performed after opening the rotator interval. If the glenoid was not treated surgically, the tendon was left intact.

Patients who underwent surgical treatment began the physiotherapy with a passive range of motion (ROM) over six weeks. During the first three weeks, passive abduction and flexion were limited to 60° and then extended to 90° for the next three weeks. For non-operative GRF management or when PHF was treated with ORIF, pendulum exercises were recommended for the first ten days, followed by passive abduction and flexion up to 90° for 4–6 weeks. The rehabilitation protocol could vary based on individual patient factors or intraoperative findings.

## Statistical methods

The mean values, percentages, and statistical differences were calculated. An unpaired t-test was used to test for statistical significance. A result was defined as significant at a value of p < 0.05.

## Results

**Fig. 3:**
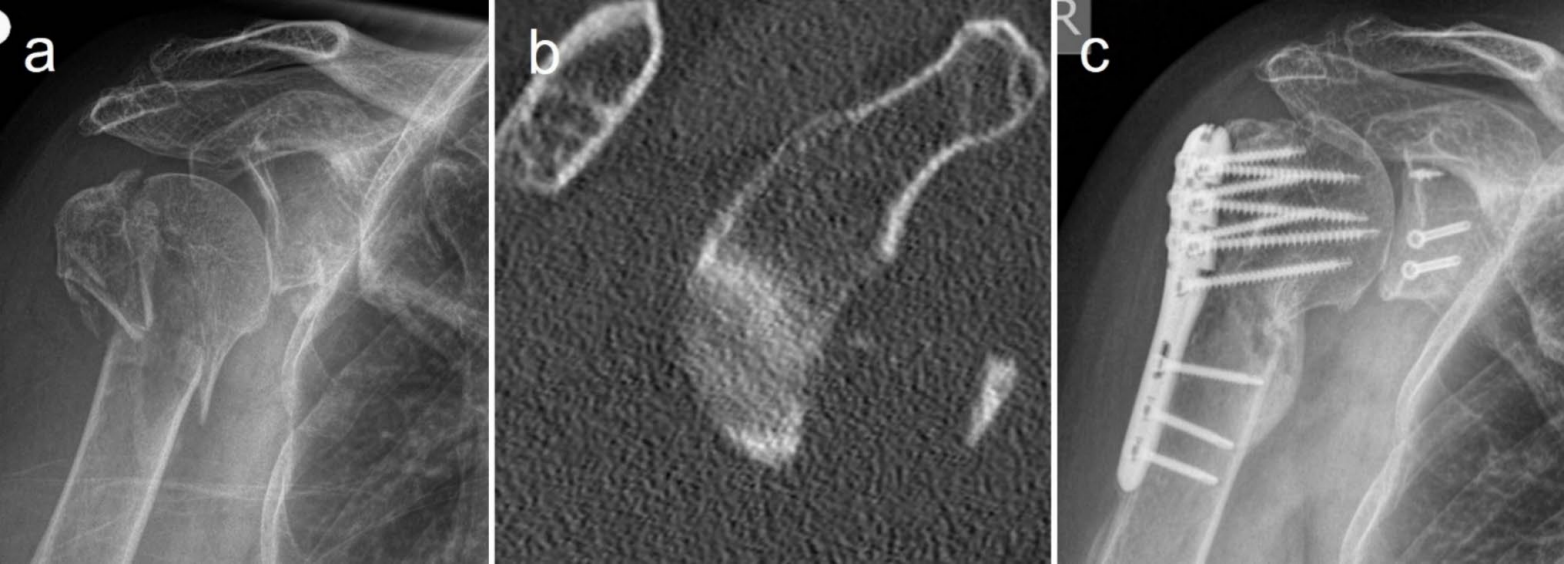
Glenohumeral combination fracture in a 72-year-old female with a large and displaced glenoid rim fracture (a.p.-radiograph, b CT scan sagittal plane) indicated for surgery. Image c (a.p.-radiograph) shows the postoperative result within further course with prolonged bony consolidation and an excellent clinical result (CS 78, WOSI 82, OXSC 41, RS 80)

A total of 27 patients with GCF type 2 were identified. Eight patients who underwent shoulder arthroplasty due to cuff arthropathy or poor bone quality were excluded, leaving 19 patients who met the inclusion criteria. Of these, three patients (16%) were lost to follow-up, resulting in 16 patients with 17 GCFs being followed up (Fig. 3). The mean age of these patients was 62 years (range: 47–82 years; ± 10 years), with seven men and nine women. The average follow-up duration was 39 months (range: 14–82 months; ± 24 months). The most common fracture type in PHF was the 3-part fracture (n = 13; 76%), followed by 4-part fractures (n = 2; 12%) and surgical neck fractures (n = 2; 12%). According to Resch et al.'s classification, 17 PHFs corresponded to type 5, with eight classified as AO C3, seven as AO B3, and two as AO A3 fractures. Twelve GRFs consisted of a single fragment, while five had comminuted glenoid rim fractures.

In two cases, the humeral head was locked at the fractured anterior glenoid rim in the initial radiographs, necessitating surgical reduction. Most patients (n = 14, 88%) underwent surgical treatment. One patient with bilateral GCF had both sides treated operatively. In six cases, the humeral head was treated with locking plate osteosynthesis, while the GRF was managed non-operatively. In nine cases, both the humeral head and glenoid rim fracture were surgically reconstructed. Two cases were managed non-operatively for both lesions. The glenoid component was never surgically fixed when the PHF was treated conservatively.

Among the follow-up patients, the average preoperative medial displacement of the GRF was 5 mm (range: 1–18 mm). Patients who underwent isolated surgical treatment of the PHF (n = 6; 35%) had an average preoperative medial displacement of 3 mm (range: 0–6 mm), while those who had both lesions surgically treated (n = 9; 53%) had an average displacement of 8 mm (range: 1–18 mm). In the sagittal plane, the average fracture displacement ("dehiscence") was 4 mm (range: 1–10 mm).

The average WOSI score in the patient group (n = 17) was 75% (range: 10–100%; ± 22.51%), the mean OSS was 41 points (range: 7–48 points; ± 10.01 points), the mean Rowe score was 77 points (range: 30–100 points; ± 21.55 points), and the CMS was 68 points (range: 23–88 points; ± 17.7). Clinically measured abduction across all patients was 138° (range: 90–180°; ± 33°) and flexion was 140° (range: 90–180°; ± 31°). The average external rotation of the affected shoulder (with the elbow flexed to 90°) was 38° (range: 10–60°; ± 16°). No clinical instability was observed in the patient group.

For surgically treated patients (excluding the two managed non-operatively), the average WOSI score was 72% (range: 10–99%; ± 22.15%), OSS was 40 points (range: 7–48 points; ± 10.4 points), Rowe score was 74 points (range: 30–100 points; ± 21 points), and CMS averaged 67 points (range: 23–88 points; ± 18.06 points). The average external rotation was 35° (range: 10–60°; ± 14°). For patients who had both GFR and PHF surgically treated, the external rotation was 32° (range: 10–60°; ± 15°). For those treated non-operatively for the glenoid, the average external rotation was 28° (range: 15–45°; ± 10°) (p > 0.05). Further details are provided in Tables 2 and 3.

**Table 2:** Statistical overview

Parameters	In total (N = 17)	Surgical therapy in total (N = 15)	Surgical therapy PHF u. GRF (N = 9)	Surgical therapy PHF (N = 6)
Age	62 y (47–82 y; ± 10 y)	62 y (47–82 y; ± 10 y)	62 y (47–75 y; ± 8 y)	63 y (47–82 y; ± 12 y)
Follow-up time	39 m (14–82 m, ± 24 m)	36 m (14–76 m, ± 23 m)	38 m (14–76 m, ± 23 m)	34 m (14–65 m, ± 21 m)
Abduktion	138° (90–180°; ± 33°)	133° (90–180°; ± 32°)	142° (90–180°; ± 31°)	120° (90–180°; ± 30°)
Flexion	140° (90–180°; ± 31°)	135° (90–180°; ± 29°)	138° (90–180°; ± 29°)	132° (90–180°; ± 28°)
External Rot	38° (10–60°; ± 16°)	35° (10–60°; ± 14°)	38° (10–60°; ± 15°)	37° (15–60°; ± 17°)
CMS	68 pt (23–88 p; ± 17,7 pt)	67 pt (23–88 pt; ± 18,06 pt)	70 p (23–88 pt; ± 14,73 pt)	61 pt (42–86 pt; ± 16,04 pt)
ROWE	77 pt (30–100 pt, ± 21,55 pt)	76 pt (30–100 pt; ± 22 pt)	77 pt (30–100 pt; ± 21 pt)	68 pt (35–100 pt; ± 20 pt)
OSS	41 pt (7–48 pt; ± 10,01 pt)	40 pt (7–48 pt; ± 10,4 pt)	40 pt (7–48 pt; ± 12,12 pt)	41 pt (30–48 pt; ± 7,01 pt)
WOSI	75% (10–100%; ± 22,51%)	72% (10–99%; ± 22,15%)	72% (10–99%; ± 25,89%)	73% (55- 91%; ± *14*,86%)

**Table 3:** Patient overview

Gender (m/f)	Age (y)	FU (m)	Class (AO)	Surgical therapy (PHF/ GRF/ None)	ROM (Abd/Flex/ER)°	CMS (pt)	RS (pt)	OSS (pt)	WOSI (%)
f	58	17	B3	PHF/GRF	160/170/30°	73	75	37	77
m	47	17	B3	PHF	180/180°/n.d	86	100	48	90
m	59	76	C3	PHF/GRF	150/120/10°	76	65	42	64
m	51	26	A3	PHF	120/130/45°	65	65	34	55
f	72	14	C3	PHF/GRF	120/120/40°	78	80	41	82
f	70	65	C3	PHF	100/120/20°	47	65	30	56
m	75	52	C3	PHF/GRF	95/110/25°	69	65	47	87
f	59	64	B3	PHF/GRF	180/180/40°	86	100	48	99
m	82	18	C3	PHF	90/90/30°	42	35	39	67
f	69	62	C3	PHF	100/120/30°	76	80	47	91
			C3	PHF/GRF	150/130/40°	79	80	47	93
f	61	19	B3	PHF/GRF	170/160/60°	88	100	47	83
f	65	45	B3	PHF/GRF	90/90/40°	23	30	7	10
f	47	14	C3	PHF/GRF	160/160/60°	62	100	43	52
f	58	14	B3	PHF	130/150/15°	50	65	47	80
m	54	82	A3	None	180/180/60°	82	100	48	99
m	72	31	B3	None	170/175/60°	82	100	46	100

**Fig. 4:**
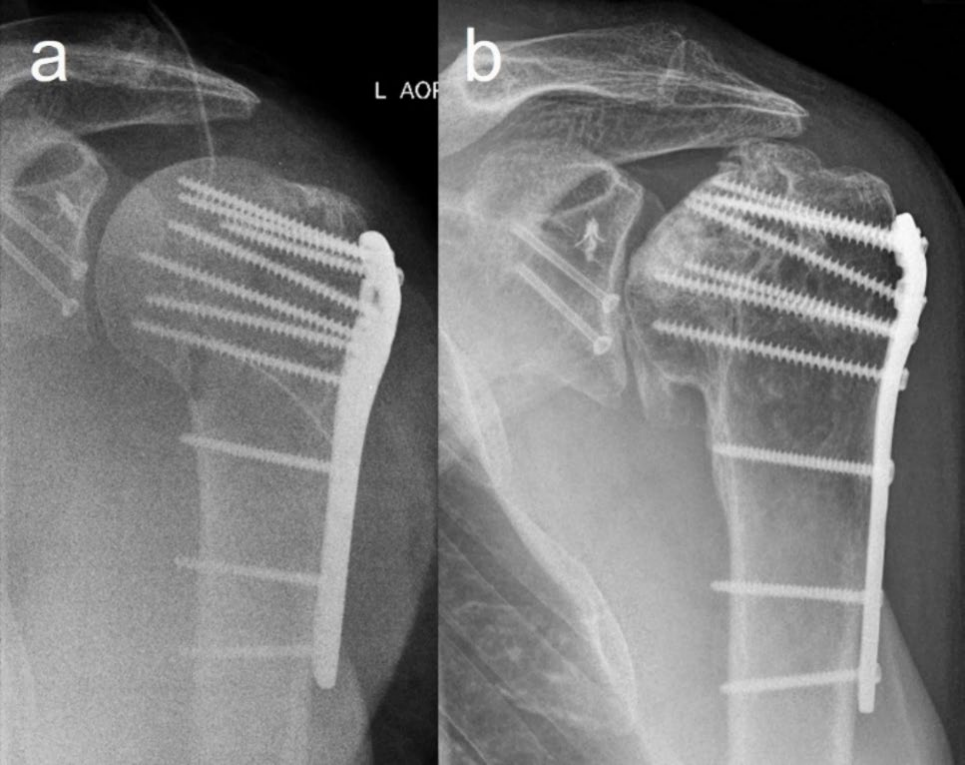
59 year old male with avascular necrosis of the humeral head 6 years after ORIF of both lesions of the glenohumeral combination fracture (a + b, a.p.-radiograph) with still moderate clinical function

The complication rate among the 17 GCFs in the follow-up group was 29% (n = 5), with complications primarily occurring on the humeral side. Avascular necrosis (AVN) of the humeral head was observed once after six years (Fig. 4), and partial necrosis of the greater tuberosity occurred in one patient. One case involved a peri-implant infection, and screw loosening on the glenoid side was seen in another case. One patient developed post-traumatic osteoarthritis, progressing from grade I to grade III (Samilson and Prieto [34]) over several years. During follow-up, only the peri-implant infection necessitated implant removal and revision surgery (6%).

Three older patients (ages 82, 75, and 65) had restricted ROM (Abduction/Flexion/External Rotation: 90/90/30°, 95/110/25°, 90/90/40°) but maintained sufficient passive ROM, so arthrolysis was not indicated.

## Discussion

The present study represents the first follow-up study of rare glenohumeral combination fractures, with reconstructive surgical management in most cases. Overall, good outcomes can be achieved with ORIF, both when fixing both lesions and when only addressing the proximal humerus fracture (PHF), leaving the glenoid rim unaltered. In most cases, however, operative treatment was indicated.

The literature provides limited data on clinical outcomes following GCF, with isolated results found only in case series or studies focused primarily on GRF. One study on GRF treatment included two patients with associated 3-part humeral head fractures involving the minor tuberosity, managed surgically using a subscapularis split approach. These patients had poorer range of motion outcomes, attributed to a more restrictive rehabilitation protocol. No re-dislocations or bone loss were observed during follow-up [29].

Another study reported on 26 patients with mixed type 1 and type 2 GCF, all treated with reverse total shoulder arthroplasty (RSA) and additional glenoid bone grafting, with a mean age of 68.5 years (63–75 years). Direct comparison with ORIF outcomes from the present study is challenging due to the mixed fracture types within the study of Garofalo [37].

Ong et al. presented a case series of three patients with combination fractures of the proximal humerus and anterior glenoid rim, with a mean follow-up of 20 months and an average patient age of 62 years (50–68 years). All patients suffered a direct impact on the affected shoulder, including one glenohumeral dislocation. Fractures included two 3-part and one 4-part, with one patient also sustaining a transmural rotator cuff tear. Surgical treatment via a deltopectoral approach was used for all, with glenoid fixation achieved through cannulated screws or anchors. Reported complications included frozen shoulder and post-operative axillary and suprascapular nerve palsy in one patient with a Neer 6 fracture. The mean CMS was 48 points [31].

Oh et al. described a middle-aged male patient with GCF following high-energy trauma treated arthroscopically. The PHF was managed with closed reduction and internal fixation under arthroscopic control, and the GRF was fixed with arthroscopic screw placement, resulting in satisfactory clinical and radiological outcomes [30].

Given the limited data on GRF in the literature, comparisons of these injuries can also be made to studies reporting on isolated GRF or PHF. Studies on isolated anterior GRF report good to excellent results with non-operative management, with low rates of re-dislocation and no clear link between fragment size or displacement and secondary instability [3–5, 20, 36, 38]. Interestingly, the incidence of osteoarthritis appears similar between non-operative and operative treatments, suggesting that initial trauma contributes significantly to osteoarthritis development in isolated GRF cases [11, 20, 24, 29, 36, 39–41].

Postoperative complications predominantly affected the humeral side, consistent with findings from other studies showing higher complication rates following PHF surgery compared to glenoid fracture surgery [8, 15, 17, 19, 20, 22, 24, 29, 40]

The prognosis for GCF is variable and influenced by fracture morphology and minor tuberosity involvement. When the minor tuberosity is involved, the GRF should be addressed first, followed by the PHF. If the minor tuberosity is intact, the PHF should be treated first, with the glenoid fragment addressed subsequently via SSC tenotomy or subscapularis-split approach.

For isolated GRF, the concentricity of the humeral head plays a minor role in surgical decision-making for type 2 GCF due to the difficulty in accurately assessing concentricity when multi-fragment PHF is present [4, 5, 36]. Decisions should be patient-specific, taking into account age, comorbidities, and other factors, with the surgical approach tailored by the treating surgeon.

Surgeons should be vigilant for additional glenoid fractures in PHF cases, avoiding a narrow focus on PHF alone to prevent missing relevant GRF. Preoperative CT scans of both lesions are crucial for accurately detecting and assessing glenoid fragment displacement and size.

We recommend reverse shoulder arthroplasty for GCF patients over 65 years with poor bone quality, rotator cuff tears, or arthropathy. For younger patients (under 40), surgical fixation of displaced glenoid fragments is advised to minimize instability risk. In middle-aged patients (over 40/45 years), surgical treatment is suggested for glenoid involvement of approximately 25% with displacement over 3 mm. Even large glenoid fragments without displacement generally do not require surgery [2, 5].

There are certain limitations to the present study: The retrospective nature of our study with varying follow-up durations, and due to the rarity of these combination fractures, the patient group is heterogeneous regarding treatment approaches.

## Conclusion

Managing GCF type 2 fractures (PHF and GRF) is challenging. Reconstruction of the PHF and/or glenoid via a deltopectoral approach is a viable alternative to shoulder replacement, especially for younger and middle-aged patients. Preoperative CT is essential for accurate fracture assessment and to ensure GRF is not overlooked. Minor tuberosity involvement significantly influences subscapularis tendon management and surgical approach selection. A careful evaluation of glenoid fragment size and displacement, alongside patient-specific factors and associated injuries, is key to optimizing surgical strategy.

## Data Availability

All data underlying the results are available as part of the article and no additional source data are required.
